# Development and validation of the Redeemer’s University Suicidality Scale

**DOI:** 10.4102/sajpsychiatry.v29i0.1799

**Published:** 2023-03-29

**Authors:** Bede C. Akpunne, Ebenezer O. Akinnawo, Abayomi O. Olusa, Daniel O. Kumuyi

**Affiliations:** 1 Department of Behavioural Studies, Faculty of Social Science, Redeemer’s University, Ede, Nigeria; 2 Department of Pure and Applied Psychology, Faculty of Social Sciences, Adekunle Ajasin University, Akungba, Nigeria

**Keywords:** development, validation, culturally suitable, suicidality scale, Nigeria

## Abstract

**Background:**

The need for a culturally suitable scale for suicidality within the multilingual Nigerian society necessitated this research interest.

**Aim:**

The study is a development and validation of the Redeemer’s University Suicidality Scale (RUSS).

**Setting:**

South western Nigeria.

**Methods:**

This comprised of initial generation of items; face and content validity, item refinement and administration of RUSS to 150 university undergraduates, using exploratory factor analysis at the first, second and third stages. In the fourth stage, 184 undergraduates responded to the 20-item RUSS, Suicide Ideation Scale (SIS) and General Health Questionnaire (GHQ-12). Data gathered at this stage were analysed for congruent validity, reliability and norms.

**Results:**

The principal component analysis extracted four components from items whose eigenvalues exceeded one. Twenty-one of the 25 items loaded best in the first, two in the second and one on the third component(s). Only items in the first component were retained. Item-total correlation further showed that the values of one item in the first component fell below the very good discrimination and was deleted from the scale. The RUSS has a Cronbach’s alpha of 0.93. Congruence validity coefficient of *r* = 0.881 (*p* < 0.001) and *r* = 0.605 (*p* < 0.001) was observed between RUSS and SIS and between RUSS and GHQ-12, respectively.

**Conclusion:**

The RUSS is gender-sensitive, has acceptable psychometric properties and is recommended as a diagnostic tool for assessing suicidal behaviour in adolescents and adults.

**Contribution:**

This article contributes to the development of a culture sensitive measure for suicidality.

## Introduction

Suicide was the 18th most prominent cause of death worldwide, accounting for 1.4% of deaths.^1^ Suicide is also one of the top 10 causes of death in the general population and the second-highest cause of mortality among people aged 15–34 years old.^2,3,4,5^ Suicide affects people of all ages. Suicide is the second leading cause of death for those aged 10–34, the fourth leading cause of death for people aged 35–54 and the eighth leading cause of death for people aged 55–64 in the United States of America.^2^ Every year, over 800 000 people die by suicide around the world, and it is estimated that for every person who dies by suicide, more than 20 others attempt suicide.^3,6^ According to statistics, the rate of suicide varies by country and location.^4^

Although suicidal behaviour and non-suicidal self-harm are related terms, the two concepts are different. While suicide is an act of escaping pain and suffering by taking one’s own life, self-injury is a non-suicidal activity used as a coping mechanism. It can provide relief from excruciating pain or emotional numbness and can last for weeks, months or even years.^7^ Non-suicidal self-harm is a behaviour that people engage in when trying to cope with negative feelings or pain. Cutting, burning, scratching, head-banging and punching walls are examples of non-suicidal self-harm. Non-suicidal self-harm involves deliberate activities designed to alleviate emotional discomfort or bad feelings. Suicidal behaviour, on the other hand, entails contemplating, planning, imagining or acting on suicidal ideas or urges. It refers to any action taken by a person to end their life.^8^ Some of the differences are the motivation behind actions, the quantity of damage, the method of self-harm, the frequency of action and so on.^8^

Suicidal behaviour encompasses a variety of behaviours that have the same goal of killing oneself. Suicidal behaviour, however, differs from non-suicidal self-injury, which is a deliberate destruction of one’s own bodily tissue with no lethal intent, although the two behaviours might lead to dying.

Suicidal ideation, planning and attempts are three essential suicidal behaviours. Suicidal ideation occurs when a person considers or plans to commit suicide.^3^ Suicidal ideation encompasses a wide spectrum of behaviours, from fleeting thoughts to long-term plans. Suicidal ideations, often known as suicidal thoughts or ideas, is a broad term that refers to a variety of thoughts, aspirations and preoccupations with death as well as the inclination or desire to terminate one’s own life.^9,10^ Suicidal ideation can range from passive (wanting to die) to active (wanting to kill oneself or thinking of a specific method for doing it).

A suicide plan is defined as ‘a proposed technique of carrying out a design that may result in self-injury; a systematic development of a plan of action that may result in self-injury’.^11^ Finally, attempting suicide is a planned act of ending one’s life. It is a self-inflicted, possibly harmful behaviour with a nonfatal outcome for which there is evidence of a desire to die (either explicit or implicit).^11^ Overdosing or ingesting is the most common means of suicide attempt among teenagers, followed by hanging or suffocation and using a sharp object (e.g. cutting).^12,13^

Different theories have been used to explain suicidal behaviour. One of them is the ideation-to-action framework proposed by Klonsky and May.^14^ According to this model, the development of suicidal ideation and the progression from suicidal ideation to attempts should be seen as distinct processes having distinct predictors and explanations. The ideation-to-action framework sheds more light on what the traditional approaches (psychache, social isolation, escape from aversive self-cognitions, hopelessness and so on) could not explain (i.e. offering different explanations for the development of suicidal ideation and the progression from ideation to attempt). The traditional differs from the ideation-to-action model in that the former treats suicide as a risk and a unitary construct. At the same time, the latter distinguishes ideation predictors from the progression from ideation to behaviour.

The ideation-to-action framework, which specifies: (1) the development of suicidal ideation and (2) the transition from ideation to suicide attempts, is one perspective in the study of suicide. Suicidal ideation, suicide attempts and suicide are all included in this framework,^15^ with suicidal ideation being the first phase of suicide. As a result, any self-initiated or committed activities with the aim or expectation of dying, including active or passive self-inflicted acts, are considered suicide.^16^

Another significant advancement is a growing collection of research distinguishing between characteristics that predict suicide thoughts and those that predict attempted suicide.^17^ According to studies, most suicidal behaviours occur when people are thinking about it.^18^ Suicidal behaviour is influenced by various biological, psychological, social, cultural and spiritual variables.

Suicidal ideation is defined as a desire or consideration of suicide.^19^ In the literature, there are two types of suicidal ideation (passive and active). Passive suicidal ideation happens when someone aspires to die or believes they might die but has no intention of actually doing so. Active suicidal ideation, on the other hand, refers to the act of thinking about, intending to and planning to commit suicide.^19^ Suicidal ideation is a significant risk factor for suicide in adolescents and young people.^20,21^ Suicidal ideation has been reported by almost a third of teenagers aged 12–20 years in the United Kingdom.^22^

Suicidal behaviour is common among those suffering from mental illnesses such as depression, schizophrenia, bipolar disorder and substance misuse.^19^ However, it is not always associated with an underlying disease or a genuine desire to die.^23^ Despite the global rise in suicide rates, Rukundo et al.^24^ found a data vacuum in suicidal behaviour among children and adolescents in poor and middle-income African nations.

Like many other countries, Nigerian research shows an alarming rate of suicide deaths.^25,26^ For instance, according to 2019 statistics, there is a 3.5 suicide rate per 100 000 Nigerians.^27^ Based on an estimated 2021 population of over 211 million people, the death by suicide figure in 2021 was about 7400.^27^ Most suicide deaths occur in underdeveloped nations such as Nigeria^17^ and are often undetected^28^ and grossly unreported.^6,29,30^ According to the World Health Organization’s (WHO) 2018 rating,^3^ Nigeria has the highest suicide rate in Africa and ranks fifth in the world in terms of acute suicide cases, trailing only South Korea, Russia, India and Japan.^31^

### Justification for the study

The rate of death by suicide is high in Nigeria.^3,25,26,32^ Nigeria, with a population of over 200 million people, has a high suicide prevalence rate.^1^ Judging from the identified perpetuating risk factors such as poverty, financial constraints, issues with relationships, loss of loved ones, family history of suicide, mental illness, substance abuse and physical illness,^32^ and the dearth of data on suicide resulting from under-reporting for fear of stigmatisation,^33^ there is a need for a standardised scale to identify suicidal behaviour among Nigerians.

This scale will help in early risk identification, reducing suicide deaths if adequate interventions are provided and practical policy declarations to address it. The need for an indigenous scale that considers the unique sociocultural elements that affect Nigerians and how these factors influence suicidal behaviour form a strong argument for this study. Based on this background, the authors set out to create and validate an indigenous suicidality scale.

## Research methods and design

### Study sample

The sample size calculation was based on the table of sample size determination published by Glenn.^34^ For a sample size for ± 10% precision levels where confidence level is 95%, and *p* = 0.5 for 4000, a population sample size of 98 was calculated.^34^ Based on a previous pilot study conducted using 180 participants, there was a 35.9% prevalence of suicidal behaviour. To account for attrition, a sample size of 150 and 184 participants who consented was used for the exploratory factor analysis (EFA) and the determination of the psychometric properties of the Redeemer’s University Suicidality Scale (RUSS), respectively.

The participants’ ages ranged between 15 and 34 years (mean = 19.72; s.d. = 3.61). A cross-sectional method was used to select participants from all levels of study in the seven faculties in the selected institution. The faculties are Natural Sciences, Social Sciences, Management Sciences, Humanities, Law, Basic Medical Sciences and Engineering.

For the inclusion and exclusion criteria, only registered students who were university undergraduates and currently on full-time study bases, found within the selected university campus, were selected for the study. This inclusion was meant to provide valid research outcomes by controlling for possible error in the responses caused by students who were not undergraduate students or students who were not on a full-time study basis but might be around the university campuses at the time of instrument administration. Also, only students willing to answer and return the questionnaires to the researcher were included in the study. This inclusion criterion enabled the researcher to ensure that all the prospective participants were well enlightened and understood the purpose of the study; as such, motivation and true responses were guaranteed.

Studies at the international^35^ and Nigerian levels^36,37^ show that the age category 18–29 years is reported to have the highest prevalence of suicidal behaviour. Also, suicide is more prevalent among undergraduates,^32^ hence choosing this population for the study. Previous studies have suggested being young as a risk factor for suicidal behaviour.^38^

### Study setting

This study was carried out among undergraduate students at a private university in south-western Nigeria.

### Study instruments

The participants responded to the RUSS, the Suicide Ideation Scale (SIS)^39^ and the General Health Questionnaire 12 (GHQ-12).^40^

The SIS, originally developed for and validated with nonclinical samples of young adults, was designed as a brief measure of ideation for use among clinical and nonclinical populations. The SIS is a 10-item self-report scale designed to assess the severity or intensity of suicide ideation. Responses on the SIS are scored on a Likert-type scale with anchors at 1 (never or none of the time) to 5 (always or a great many times) based on how the respondent has felt or behaved over the past year. Items in the SIS are:

- I have been thinking of ways to kill myself.
- I have told someone I want to kill myself.
- I believe my life will end in suicide.
- I have made attempts to kill myself.
- I feel life just isn’t worth living.
- Life is so bad I feel like giving up.
- I just wish my life would end.
- It would be better for everyone involved if I were to die.
- I feel there is no solution to my problems other than taking my own life.
- I have come close to taking my own life.

Items were scored by direct scoring, and the total score was calculated by finding the sum of 10 items. The total score ranges from 10 to 50. Rudd^39^ reported a high internal consistency (Cronbach’s alpha of 0.86) and adequate item-total correlations (*r* = 0.45–0.74). The SIS was moderately correlated with the Centre for Epidemiology Studies – Depression Scale (*r* = 0.55) and the Beck Hopelessness Scale (*r* = 0.49). The SIS has been used in Nigerian studies. For instance, Nkwuda, Ifeagwazi, Nwonyi and Oginyi^41^ reported a Cronbach’s alpha of 0.90 among Nigerian undergraduates, while Okeke and Ogbonnaya^42^ reported a Cronbach’s alpha of 0.89 among Nigerian prison inmates.

The GHQ-12 was designed to assess psychological distress.^40^ The 12 items of the GHQ-12 are scored on a 4-point severity and frequency scale (0–3). Scores of the items of the GHQ-12 are added to derive the total score of psychological distress. The GHQ-12 has acceptable psychometric properties for the Nigerian population.^43^ As a measure of psychological distress,^40^ the GHQ-12 was used to validate suicidality, which is also a manifestation of psychopathology. The researchers collected data for the study.

#### Statistical analysis

Data were analysed using the Statistical Package for the Social Sciences (SPSS) version 23 (IBM Corporation, Armonk, New York, United States). Factor analysis was used for the exploratory factor analysis (EFA). Reliability analysis was used to find out the Cronbach’s alpha, Guttman split-half and Spearman–Brown coefficients. Finally, Pearson correlation analysis was used to determine the congruence validity of the scale under development.

### Results

#### Item generation for the Redeemer’s University Suicidality Scale

**FIGURE 1:**
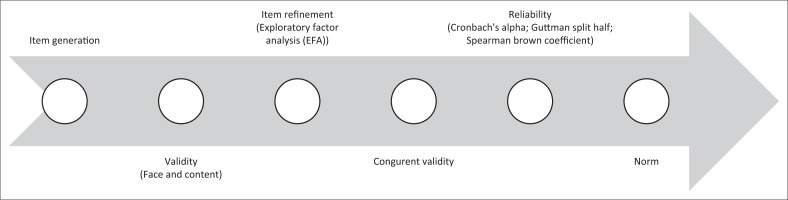
Flow diagram for the suicidality scale development and validation.

Figure 1 is a flow chart depicting the steps taken in developing and validating the RUSS. The steps include item generation, face and content validity, Item refinement, congruent validity, reliability and norms of the RUSS. The items for the RUSS were based on the symptoms of suicidality (suicide ideation, planning and attempt) found in both the Diagnostic and Statistical Manual of Mental Disorders, 5th edition (DSM-5), and the International Classification of Diseases, 10th revision (ICD-10).^44,45,46^

Thirty-seven items were generated using three of the sources recommended by Furr (2011) and Streiner (2015). These sources are an examination of items in some instruments that measure suicide, a review of theories of suicide for potential ideas and research findings of previous studies. Clinical features of depression and suicidality in the DSM-5 were also considered.^44,45,46^

### Validity of the Redeemer’s University Suicidality Scale

The generated items were subjected to both face validity and content validity assessment by a panel of experts made up of one psychiatrist and six clinical psychologists. The experts reviewed items for content regarding their relevance to the content domain, completeness and accuracy. They also reviewed for item sensitivity to evaluate potential item bias.^47^ The items were evaluated using the content validity ratio (CVR) of a four-point scale of 1 = not relevant, 2 = somewhat relevant, 3 = quite relevant but needs rewording and 4 = highly relevant.^48^ All items checked as ‘not relevant’ were eliminated. Using 75% item inclusion (i.e. 75% of the experts must approve an item for inclusion), those checked as ‘needs rewording’ were reworded, and items checked as highly relevant were retained. Thirty items were retained after CVR. Lastly, the 30 items were re-presented to the experts for review on a two-point response format of ‘yes’ and ‘no’. Items checked as ‘yes’ were included while those checked as ‘no’ were removed. Using the 75% item inclusion, 25 items were retained. The justification for employing the face validity method was that the expert technique is acceptable for content validity^49^ when combined with a comprehensive identification process as outlined above. This process resulted in a final version of 25 valid items used for item refinement.

### Item refinement

One hundred and fifty university undergraduates (male, 79; female, 71) were purposively drawn from a Nigerian private university. A cross-sectional method was used to select participants from all levels of study in the seven faculties in the chosen institution. The faculties are Natural Sciences, Social Sciences, Management Sciences, Humanities, Law, Basic Medical Sciences and Engineering. The responses of these participants were used for the EFA (item refinement) of the RUSS. The participants’ ages ranged between 15 and 34 years (mean = 19.72; SD = 3.61).

The 25 items of the RUSS were subjected to EFA. Factors with eigenvalues > 1 were extracted at the first stage of EFA. Next, the statistics for factors with eigenvalues > 1 were scrutinised. Stevens^50^ recommended 0.40 as the least factor loading. Items having loadings of less than 0.45 were deleted to improve the RUSS interpretability. The different plausible factor solutions were evaluated considering the items’ content and the proportional construct of interest.

### Exploratory factor analysis

According to Pallant^51^ for factor analysis to be considered appropriate, Bartlett’s test of sphericity (BTS) should be significant (*p* < 0.05), with 0–1 range for the Kaiser–Meyer–Olkin (KMO) index and 0.06 set as the minimum value for suitable factor analysis. The results of the tests are presented in Table 1.

**TABLE 1:** Summary of Kaiser–Meyer–Olkin and Bartlett’s Test of Sphericity on the factorability of the 25-item measure for the Redeemer’s University Suicidality Scale.

Measurement	Values
**Bartlett’s Test of Sphericity (BTS)**	
Approximate chi-square	3507.995
df.	351
Sig.	0.000
KMO measure of sampling adequacy	0.93

KMO, Kaiser–Meyer–Olkin; BTS, Bartlett’s Test of Sphericity; df, degrees of freedom; Sig., significance.

Table 1 indicates that the KMO measure of sampling adequacy is 0.93, and it is within the recommended range of 0–1. The BTS is significant (*X*^2^ = 3507.995, degrees of freedom [*df*] = 351, *p* = 0.000). Therefore, the results support the correlation matrix’s factorability, and hence the principal component analysis (PCA) is conducted. The principal component extraction method’s test indicated four components extracted with eigenvalues > 1, and the summary is presented in Table 2.

**TABLE 2:** Total variance explained.

Components	Eigenvalues	% of variance	Cumulative %
1	14.018	51.918	51.918
2	1.796	6.647	58.566
3	1.489	5.513	64.079
4	1.021	3.782	67.861

Table 3 summarises the principal component matrix analysis showing the extracted four components with eigenvalues above 1 for the 25-item measure for the RUSS. The loading of the 25 items under the four components is presented in Table 3.

**TABLE 3:** Component matrix of 25 items of the Redeemer’s University Suicidality Scale.

Component matrix†	Component			
	1	2	3	4
32. I told someone of my plans to kill myself	0.919	-	-	-
33. I had tried to kill myself	0.898	-	-	-
31. I looked for a perfect opportunity to execute my plan for killing myself	0.894	-	-	-
24. I feel so lonely and unwanted that I want to kill myself to end my misery	0.875	-	-	-
23. The only option out of my problems is to kill myself	0.875	-	-	-
30. I have made plans of how to kill myself	0.874	-	-	-
34. I visited online websites to find suitable ways to kill myself	0.870	-	-	-
2. I wished I were dead	0.854	-	-	-
29. I am writing or have written a suicide note	0.831	-	-	-
18. I see myself as a failure and wish to kill myself	0.827	-	-	-
25. By and large, life has been cruel to me, so I want to end it myself	0.822	-	-	-
1. I have thoughts of killing myself	0.817	-	-	-
8. Life has been unfair; I feel like giving up	0.817	-	-	-
3. I told someone my thought of killing myself	0.800	-	-	-
9. I believe my family would be better off if I were dead	0.788	-	-	-
6. I am sure I will one day kill myself	0.763	-	-	-
12. The only solution I see to my problems is suicide	0.725	-	-	-
26. I desire to die	0.698	-	-	-
20. I do not want to die	−0.688	-	-	-
35. I do not think killing myself would solve my problems	−0.558	-	-	-
4. The future looks bright for me	-	−0.817	-	-
27. Cultural rituals for death by suicide are what is keeping me from killing myself	0.483	0.490	-	-
16. I am sure that I have the will to survive	-	-	0.792	-
28. My religious belief is what is keeping me from ending my life	-	-	0.466	−0.618
5. I consider suicide morally wrong	-	-	0.471	0.563

Note: Extraction method is the principal components analysis (PCA).

PCA, principal component analysis.

† 4 components extracted.

The four components extracted summarised in Table 3 were because the items loaded on these four components with eigenvalues exceeding 1. The eigenvalues of the four components range from 14.018 to 1.021, with a percentage ranging from 51.918 to 3.782. However, only one dimension was retained, as items in the other three components either loaded more than once, rendering them complex structures or standalone items. The eigenvalues of the included component explained a total of 51.9% of the total variance.

### Congruent validity of the Redeemer’s University Suicidality Scale

#### Participants

Using a cross-sectional method, a fresh sample of 184 purposively selected undergraduates (male, 70; female, 114) were sampled. The participants’ ages ranged between 15 and 34 years (mean = 19.72; s.d. = 3.61). Participants were selected from all levels of study in the faculties of Natural Sciences, Social Sciences, Management Sciences, Humanities, Law, Basic Medical Sciences and Engineering. Data collected were used to determine the psychometric properties of the RUSS.

The RUSS was validated using the congruent validity technique to ascertain its relationship with existing measures: the SIS^39^ and the GHQ-12.^40^ Significant positive correlation coefficients were observed between the RUSS and the SIS (*r* = 0.881, *p* = 0.000) and between the RUSS and the GHQ-12 (*r* = 0.605, *p* = 0.000). This finding made the RUSS valid as a diagnostic tool for measuring suicidality among the general Nigerian population.

### Reliability of the Redeemer’s University Suicidality Scale

#### Participants

A fresh sample of 182 university undergraduates (male, 89; female, 93) was purposively drawn from a Nigerian private university. The participants’ ages ranged between 16 and 31 years (mean = 19.72; s.d = 3.61). A cross-sectional method was used to select the participants from all levels of study in the seven faculties in the chosen institution. The responses of these participants were used to ascertain the reliability of the scale. The 20 items of the RUSS that were extracted from EFA in the item refinement process were administered to the participants. Data were analysed using the SPSS.

### Ethical considerations

This study relied on human subjects for its investigation. As a result, the Helsinki Declaration was followed in terms of research ethics for human beings. The research purpose was assessed by Redeemer’s University’s Internal Research Ethics Committee, which advised protocols. The ethical requirements for this type of research are not applicable (National Code of Health Research Ethics; Nigerian National Health Research Ethics Committee) (NHREC). Section B, item A. http://www.nhrec.net/nhrec/NCHRE10.pdf

## Results

Values of the corrected item-total correlations (point-biserial) indicated discriminations in the items of the RUSS. Values between 0 and 0.19 imply poor discrimination, 0.2 and 0.39 indicate good discrimination, while ≥ 0.4 suggests very good discrimination. As observed in Table 4, the item with a value between 0 and 0.19 in the RUSS is ‘I do not think killing myself would solve my problems’ (0.12). The observed values of the point-biserial suggest that items with values below the very good discrimination should be deleted from the scale, as this could indicate an ambiguous and confusing item to participants.

**TABLE 4:** Item-total statistics of the Redeemer’s University Suicidality Scale.

Reliability statistics				
Cronbach’s alpha 0.93	*N* of Items 19			
Item-total statistics	Scale mean if item deleted	Scale variance if item deleted	Corrected item-total correlation	Cronbach’s alpha if item deleted
I have thoughts of killing myself	33.0543	268.686	0.682	0.935
I wished I were dead	33.1848	267.583	0.792	0.933
I told someone my thought of killing myself	33.2717	272.942	0.674	0.935
I am sure I will one day kill myself	33.5870	281.359	0.708	0.935
Life has been unfair; I feel like giving up	32.7391	271.932	0.675	0.935
I believe my family would be better off if I were dead	33.2283	270.756	0.758	0.934
The only solution I see to my problems is suicide	33.5978	279.936	0.759	0.935
I see myself as a failure and wish to kill myself	33.4348	273.996	0.779	0.934
I do not want to die	33.1957	284.901	0.431	0.940
The only option out of my problems is to kill myself	33.8043	286.672	0.710	0.936
I feel so lonely and unwanted that I want to kill myself to end my misery	33.3261	269.642	0.799	0.933
By and large, life has been cruel to me, so I want to end it myself	33.5761	277.994	0.777	0.934
I desire to die	33.3913	272.043	0.792	0.933
I am writing or have written a suicide note	33.5109	280.404	0.617	0.936
I have made plans of how to kill myself	33.2935	274.963	0.654	0.936
I looked for a perfect opportunity to execute my plan for killing myself	33.5217	279.180	0.628	0.936
I told someone of my plans to kill myself	33.5435	279.637	0.610	0.937
I had tried to kill myself	33.3696	276.497	0.635	0.936
I visited online websites to find suitable ways to kill myself	33.7826	289.592	0.485	0.938
I do not think killing myself would solve my problems	33.0652	297.635	0.126	0.947

Table 4 further shows the Cronbach’s alpha and the item-total statistics of the 20-item RUSS. The scale achieved a reliability coefficient of 0.93, a Guttman split-half reliability of *r* = 0.825 and a Spearman–Brown coefficient of *r* = 0.828.

### Calculation of norms for the Redeemer’s University Suicidality Scale

The cut-off points for the RUSS were determined using the 95% confidence interval (CI) method. As summarised in Table 5, with 95% confidence, the group population mean was between 32.6 and 37.7, based on 184 samples (35.13 [95% CI 32.6–37.7]); the male population mean was between a range of 27.6 and 34.4, based on 70 samples (30.97 [95% CI 27.6–34.4]); while the derived mean for the female population was between 34.2 and 41.1, based on 114 samples (37.68 [95% CI 34.2–41.1]). The group and gender categories mean score plus one standard deviation were used to calculate the cut-off points. The result showed ≥ 53, ≥ 46 and ≥ 56 as cut-off points (norm) for the group, male and female samples, respectively.

**TABLE 5:** The 95% confidence interval of cut-off point determination for the Redeemer’s University Suicidality Scale by sex.

Statistical test	Group sample	Individual male	Individual female
Margin of error	2.53	4.39	3.44
Sample size	184	70	114
Sample mean	35.13	30.97	37.68
Standard deviation	17.52	14.54	18.72
95% CI	32.6–37.7	27.6–34.4	34.2–41.1
Cut-off point	≥ 53	≥ 46	≥ 56

CI, confidence interval; RUSS, Redeemer’s University Suicidality Scale.

The final draft of the validated RUSS and the scores’ interpretations are itemised in Table 6 and Table 7, respectively.

**TABLE 6:** The final draft of validated Redeemer’s University Suicidality Scale.

Items	SD	MD	N	MA	SA
1. I have thoughts of killing myself	1	2	3	4	5
2. I wished I were dead	1	2	3	4	5
3. I told someone my thought of killing myself	1	2	3	4	5
4. I am sure I will one day kill myself	1	2	3	4	5
5. Life has been unfair; I feel like giving up	1	2	3	4	5
6. I believe my family would be better off if I were dead.	1	2	3	4	5
7. The only solution I see to my problems is suicide	1	2	3	4	5
8. I see myself as a failure and wish to kill myself	1	2	3	4	5
9. I do not want to die†	1	2	3	4	5
10. The only option out of my problems is to kill myself	1	2	3	4	5
11. I feel so lonely and unwanted that I want to kill myself to end my misery	1	2	3	4	5
12. By and large, life has been cruel to me, so I want to end it myself	1	2	3	4	5
13. I desire to die	1	2	3	4	5
14. I am writing or have written a suicide note	1	2	3	4	5
15. I have made plans of how to kill myself	1	2	3	4	5
16. I looked for a perfect opportunity to execute my plan for killing myself	1	2	3	4	5
17. I told someone of my plans to kill myself	1	2	3	4	5
18. I had tried to kill myself	1	2	3	4	5
19. I visited online websites to find suitable ways to kill myself	1	2	3	4	5

Note: Instruction: The answer key for the questions can be interpreted as follows 1 = Strongly Disagree (SD) 2 = Mildly Disagree (MD), 3 = Neutral (N), 4 = Mildly Agree (MA) and 5 = Strongly Agree (SA)

† , item is reverse scored.

**TABLE 7:** Interpretation of Redeemer’s University Suicidality Scale scores.

Scoring	Group sample	Individual male sample	Individual female sample
Normal	0–18	0–17	0–19
Mild suicidal behaviour	19–52	18–45	20–56
Moderate suicidal behaviour	53–69	46–60	57–75
Severe suicidal behaviour	70 and above	61 and above	75 and above

Item 9 (with ‘?’ symbol) is reverse scored. The total score is arrived at by adding up the items’ scores. The higher the score, the greater the severity of suicidal behaviour.

Table 7 is a summary of the interpretation of the scores based on group and individual samples. The individual samples are categorised by gender.

## Discussions

This study is a development and validation of the RUSS. The scale measures suicidal behaviours (suicidal ideation, suicide planning and suicide attempts) among the general population. From gap identification to norming, seven steps were used in the development process of the RUSS. Different sources had proposed various steps in the scale development process. For instance, Lynn^52^ proposed a two-stage strategy. The first step included generating an initial pool, and the second stage involved validation (evaluation of the instrument’s item performance). Crocker and Algina^53^ and Price^54^ both proposed 11 steps; Furr^55^ proposed five steps; Streiner et al.^56^ proposed seven steps, while DeVellis^57^ proposed eight steps. Four main features are common in most of these studies: item generation, item refinement, reliability studies and validity studies. The development process of the RUSS followed these steps.

The DSM-5 and the ICD-10 were used to review clinical aspects of suicidal behaviour (ideation, intent and planning).^44,45,46^ The generation of items relating to the agreed themes resulted in 37 items used for scale purification purposes. Because of the RUSS’s ability to measure opinions, beliefs and attitudes, a six-point Likert scale was utilised. In other words, each RUSS item is a declarative statement, hence the decision to employ a Likert answer format.^57^

The RUSS purification was carried out using a combination of reliability analysis and EFA, as proposed by Flynn and Pearcy^58^ and Pecheux and Derbaix.^59^ A team of specialists checked the content validity of the initial items generated by the authors. According to Streiner et al.,^48^ content validity represents current knowledge in the construct of interest. It is also a vital sign of an instrument’s validity and shows how viable and practicable it is.^60^

The development of the RUSS provided a foundation for further investigation of its validity and reliability. Cronbach’s alpha was 0.93, and item-total correlation was 0.13–0.79. This finding implies a good item inter-relatedness, unidimensionality and homogeneity of the construct^61,62^ among the Nigerian population. To put it another way, the Cronbach’s alpha, Spearman–Brown coefficient and Guttman split-half coefficient scores were not too high to make some items redundant.^63,64^

In conclusion, the high alpha score indicates that the RUSS has a high level of reliability. The RUSS was verified using the concurrent validity approach, as Cronbach and Meehl^65^ advised. Two standardised scales, one each for assessing suicidal ideation and psychological distress in the general population, were positively linked with the RUSS. The RUSS is a suitable measure of suicidal behaviour for adolescents and adults in Nigeria and other climes with similar sociocultural circumstances, based on its EFA and acceptable psychometric qualities.

## Conclusion and recommendations

The RUSS was created by extracting a single-factor scale with 20 items through initial item generation, expert appraisal (face and content validity) of the initial pool of items and EFA for item purification. Internal consistency (reliability coefficient) of the RUSS items is acceptable. The SIS and the GHQ-12 had strong positive correlations with the RUSS, indicating a satisfactory congruent validity coefficient.

Based on the findings of this study, the 19-item RUSS showed good internal consistency and validity scores to measure suicidal behaviour. This analysis indicates that the RUSS is reliable and valid for the Nigerian population. Norms for the group and individual (male and female) samples are indications that the scale is gender-sensitive and can be self-administered both individually and in group studies. Therefore, it is recommended as a diagnostic tool for suicidal behaviour among adolescents and adults in clinical settings. It can also be used to measure suicidality in group research settings to provide information on suicidal behaviour among the general public to enable policymaking in mental healthcare domains. Again, taking into cognisance the Nigerian sociocultural setting in its item generation and development, the RUSS is recommended not only for Nigerian use but also for use in countries with similar sociocultural situations.

## Limitations of the study

This study was conducted in the context of the Nigerian population’s distinct psychosociocultural environment. Without scale revalidation, generalising the findings and using this scale on other populations with different sociocultural traits should be addressed with caution.
